# Applications of CRISPR Technologies in Forestry and Molecular Wood Biotechnology

**DOI:** 10.3390/ijms252111792

**Published:** 2024-11-02

**Authors:** Hieu Xuan Cao, David Michels, Giang Thi Ha Vu, Oliver Gailing

**Affiliations:** 1 Forest Genetics and Forest Tree Breeding, University of Göttingen, 37077 Göttingen, Germany; xuanhieu.cao@uni-goettingen.de (H.X.C.);; 2 Center for Integrated Breeding Research (CiBreed), University of Göttingen, 37075 Göttingen, Germany

**Keywords:** CRISPR/Cas9, forest trees, genetic engineering, pathogen resistance, abiotic stress, tree breeding, genome editing, climate resilience

## Abstract

Forests worldwide are under increasing pressure from climate change and emerging diseases, threatening their vital ecological and economic roles. Traditional breeding approaches, while valuable, are inherently slow and limited by the long generation times and existing genetic variation of trees. CRISPR technologies offer a transformative solution, enabling precise and efficient genome editing to accelerate the development of climate-resilient and productive forests. This review provides a comprehensive overview of CRISPR applications in forestry, exploring its potential for enhancing disease resistance, improving abiotic stress tolerance, modifying wood properties, and accelerating growth. We discuss the mechanisms and applications of various CRISPR systems, including base editing, prime editing, and multiplexing strategies. Additionally, we highlight recent advances in overcoming key challenges such as reagent delivery and plant regeneration, which are crucial for successful implementation of CRISPR in trees. We also delve into the potential and ethical considerations of using CRISPR gene drive for population-level genetic alterations, as well as the importance of genetic containment strategies for mitigating risks. This review emphasizes the need for continued research, technological advancements, extensive long-term field trials, public engagement, and responsible innovation to fully harness the power of CRISPR for shaping a sustainable future for forests.

## 1. Introduction

Forests and perennial woody trees are essential foundations of global ecosystems, playing a critical role in mitigating climate change by acting as carbon sinks [1], providing essential resources such as timber, fuelwood, and non-timber forest products [2], and maintaining biodiversity by providing habitats for many species [3]. However, these vital ecosystems are facing a growing number of threats, including emerging pathogens [4], increasing abiotic stresses driven by climate change [5], and rising demands for forest products [6]. The rapid pace of environmental change is exceeding the natural adaptive capacity of many tree species in certain regions [7,8,9] (that is, of the globally estimated more than 17,000 tree species [10]), leading to widespread forest decline [11,12,13] and jeopardizing the crucial ecological, economic, and social services they provide. This combination of threats demands urgent action and innovative solutions to safeguard the long-term health and resilience of forests worldwide.

Traditional tree breeding, while valuable for enhancing desirable traits like growth rate, wood quality, and pest resistance, is inherently a slow and laborious process [14,15,16,17,18,19]. This is primarily due to the long generation times of trees, which can span decades for many species, making it challenging to select and propagate superior genotypes within a reasonable timeframe [20,21,22]. Furthermore, the complex genetic architecture of many important traits, often involving multiple genes with small individual effects, further complicates traditional breeding efforts [23,24,25]. Fortunately, new genomic technologies hold immense potential for addressing these challenges. CRISPR technology, originally discovered as an adaptive immune system in bacteria [26,27,28], has revolutionized genetic engineering by enabling precise and efficient genome editing in diverse organisms, including trees [29,30,31,32,33]. CRISPR represents a powerful solution for enhancing desirable traits and accelerating breeding programs [34,35].

This review explores the transformative potential of CRISPR for engineering climate-resilient and productive plantations, delving into its applications, challenges, and prospects. Beginning with an overview of various CRISPR systems, we then focus on specific applications for enhancing forest resilience, including resistance to biotic and abiotic stresses; improved wood properties; and accelerated growth. We also highlight the challenges associated with CRISPR implementation in trees and discuss potential solutions, such as advances in reagent delivery and plant regeneration. Finally, we explore the broader applications of CRISPR in forestry, such as diagnostics and gene drive, emphasizing the need for responsible innovation and long-term field trials.

## 2. CRISPR Technologies: A New Era for Forest Genomics and Breeding

### 2.1. CRISPR/Cas: The Foundation of Genome Editing

**Figure 1:**
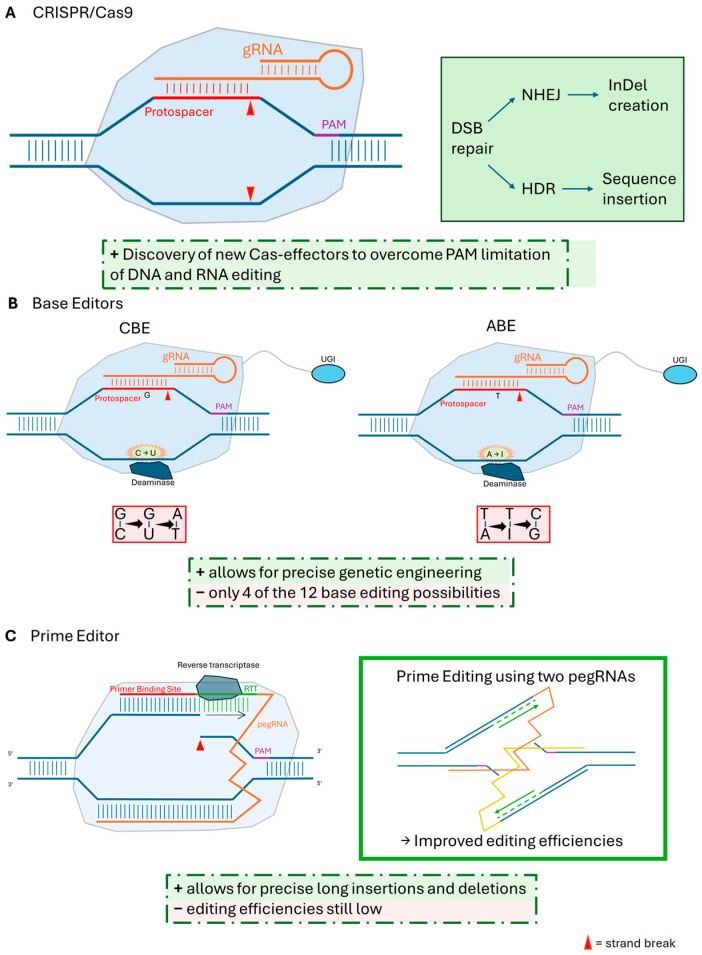
CRISPR/Cas genome editing systems. (**A**) CRISPR/Cas9: The gRNA guides Cas9 to the target locus, where Cas9 creates a DSB. The DSB can be repaired by NHEJ, often leading to InDel or HDR, which can be harnessed for precise gene editing. (**B**) Base Editors (BEs): BEs fuse a deactivated Cas9 (dCas9) to deaminase, allowing for precise single-base changes without DSBs. CBEs convert cytosine (C) to thymine (T), while ABEs convert adenine (A) to guanosine (G). Enhanced BEs incorporate a UGI (Uracil DNA glycosylase inhibitor) and a nickase Cas9 to further improve editing efficiency. (**C**) Prime Editors (PEs): PEs utilize a Cas9 nickase fused to a reverse transcriptase (RT) and are guided by a pegRNA that contains both targeting and editing information. The RT copies the edit from the pegRNA template and integrates it into the genome. Dual-pegRNAs targeting opposite DNA strands can further enhance editing efficiency. gRNA, guide RNA; PAM, protospacer adjacent motif; DSB, double strand break; NHEJ, non-homologous end joining; CBE, cytosine base editor; ABE, adenosine base editor; UGI, Uracil DNA glycosylase inhibitor; RTT, reverse transcriptase template; pegRNA, prime editing guide RNA.

CRISPR/Cas systems have revolutionized genetic engineering. These systems harness the natural defense mechanisms of bacteria to create targeted DSB in DNA [26]. The most widely employed system for genome editing utilizes the *Streptococcus pyogenes* Class 2 Type II Cas9 effector [36]. The CRISPR/Cas9 system utilizes a guide RNA (sgRNA), designed to match a specific target DNA sequence known as the protospacer, to direct the Cas9 enzyme to that location in the genome, creating a DSB (Figure 1A). This break triggers the cell’s DNA repair machinery, which can be exploited, depending on the cell cycle stage, genetic background and the surrounding DNA sequence, to introduce targeted mutations. The most common repair pathway, NHEJ, often introduces small insertions or deletions (InDels), leading to gene knockouts [29,37]. Alternatively, HDR uses a template DNA sequence to repair the break, allowing for the introduction of specific changes, such as gene insertion or replacement [38]. However, HDR-mediated editing is often less efficient.

In addition to Cas9, multiple other Cas-effectors have been discovered and characterized, significantly expanding the CRISPR toolbox [39]. These include Class 2 Type V Cas12a and the Class 2 Type VI Cas13a [40,41], which offer advantages like simplified multiplex, expanded target ranges [42], and the ability to edit RNA (Cas13a, [41]). Furthermore, a growing collection of Cas9 orthologs with unique PAM specificities and editing efficiencies [43] are providing a wider selection of tools for targeted genome editing in trees.

The growing knowledge of tree genomic sequences is crucial for various stages of the genetic engineering process, including identifying suitable target sites, predicting and mitigating potential off-target effects, verifying successful edits, and validating the functional consequences of those edits on gene function or associated traits [44]. Advanced NGS-based platforms, such as Tracking-seq, which exploits the genome-wide binding of replication protein A (RPA), a protein involved in almost all DNA damage repair processes, combined with NGS, offer direct detection of off-target effects of CRISPR editing, further enhancing precision and safety [45]. The emergence of long-read sequencing technologies, also known as third-generation sequencing [46], offers the detection of a broader spectrum of CRISPR-induced genomic alterations, including very large deletions or insertions spanning several kbp [47,48,49,50].

### 2.2. Base Editing: Precision for Targeted Single-Nucleotide Changes

Base editors (BEs) enable precise single-base changes without introducing DSBs [51]. Currently, two main types of BEs (Figure 1B) are widely used: cytosine base editors (CBEs), which convert cytosine (C) to thymine (T) [52], and adenosine base editors (ABEs), which convert adenine (A) to guanine (G) [53]. Initial limitations in efficiency and off-target effects have been addressed through continuous development, as exemplified by the refined BE3 system, which significantly enhanced CBE performance [52]. Ongoing research is expanding the targeting range, efficiency, and specificity of BEs through the development of new deaminases and Cas9 variants [54]. Furthermore, the saturated targeted endogenous mutagenesis editor (STEME) combines both cytosine and adenosine deaminase functionalities, allowing for the creation of both C->T and A->G transitions within a single system [55,56]. While the initial focus of base editing centered on transitions (A:T to G:C and C:G to T:A), recent research has expanded the repertoire to include transversions (C to A and C to G) [57,58]. These advancements further broaden the scope of base editing, offering even greater precision and versatility for engineering desired genetic changes in trees.

Base editing approaches have been widely adopted in crop and model plant research, with numerous studies demonstrating their efficacy in various species such as tomato, rice and wheat [59]. In contrast, the application of base editing in forest trees is relatively recent. Pioneering work by Li and colleagues demonstrated the feasibility of base editing in poplar hybrids, achieving high editing efficiencies using both ABE and CBE systems [60]. Specifically, they reported successful editing using two CBE and two ABE systems, with sgRNA expression driven by the AtU3 promoter. Subsequently, Yao and colleagues further confirmed the efficacy of CBE in poplar, utilizing the pHEE901-BE3 system [61]. These promising results underscore the potential of base editing for precise genome modification in woody plants.

### 2.3. Prime Editing: Versatility for Diverse Genome Modifications

Prime editing (PE) represents the latest advancement in CRISPR technology, offering unparalleled flexibility and precision in genome editing. PE enables a wider range of modifications, including all possible base substitutions, targeted insertions, and deletions without requiring DSBs or donor DNA templates [56]. The system relies on a Cas9 nickase fused to a reverse transcriptase, guided by a prime editing guide RNA (pegRNA). It carries both targeting and editing information [62]. Initial studies in rice and wheat demonstrated the feasibility of PE in plants [63]. Subsequent research has focused on optimizing editing efficiencies through strategies such as refining pegRNA design and utilizing dual-pegRNA systems (dual-pegRNAs), which utilize two pegRNA targeting opposite RNA strands to further improve editing efficiencies (Figure 1C) [64]. The targetable range has also been expanded by using Cas9 variants like SpG Cas9, which recognize a wider range of PAM sequences [64]. A web application, PlantPegDesigner (http://www.plantgenomeediting.net/), has been developed to assist researchers in designing effective pegRNAs and dual-pegRNAs for plant applications.

In mammalian cells, PE has achieved remarkable potential, including large deletions (up to 10 kb) and insertions (up to 250 bp) [65] and even the insertion of DNA sequences up to 36 kb by employing a strategy that combines prime editing with serine integrase-mediated integration [65,66,67]. These advancements highlight the transformative potential of PE for precise and complex genome engineering in diverse organisms, including woody plants.

## 3. Applications of CRISPR Technologies in Forest Tree Improvement

During their long lifetime, forest trees are exposed to a multitude of biotic and abiotic stressors. Climate change is exacerbating these challenges, intensifying the frequency and severity of droughts, storms, and temperature extremes [68], while also creating conditions that favor the spread of pathogens and pests [13]. Concurrently, the global demand for wood-based products continues to rise [69], underscoring the need for productive and resilient forests.

**Figure 2:**
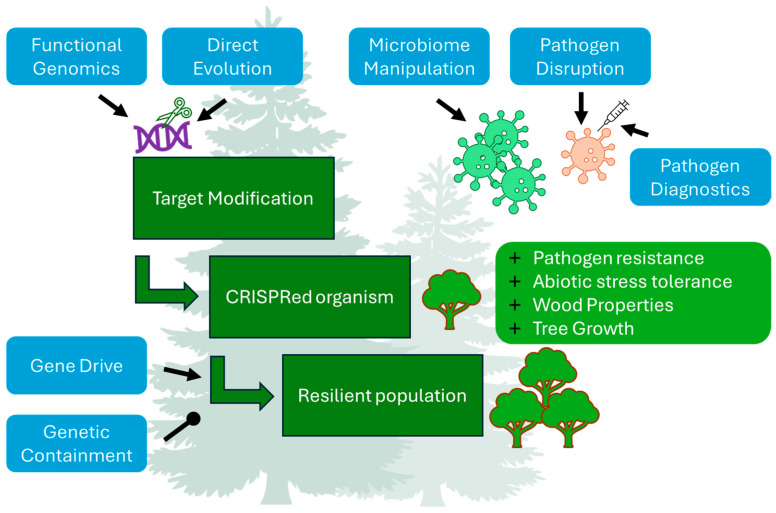
Diverse applications of CRISRP technologies in forestry. CRISPR technology offers a diverse array of applications for engineering climate-resilient and productive forests, including enhancing resistance to biotic and abiotic stresses, improving wood properties, and accelerating tree growth. Targeted modifications can be identified using CRISPR-based functional genomics (studying gene function through disruption or modification) and direct evolution (generating genetic diversity and selecting for improved or novel traits). Beyond engineering trees themselves, CRISPR systems can target DNA or RNA of viral, bacterial, fungal, and oomycete species for diagnostics, pathogenicity research, or microbiome manipulation. CRISPR gene drive technology facilitates the spread of beneficial genetic modifications through plantations, while CRISPR-based genetic containment strategies help prevent the spread of engineered genes to wild populations, promoting responsible innovation.

Breeding and genome editing offer promising avenues for enhancing desirable traits in forest trees, such as resistance to biotic and abiotic stresses, improved wood properties, and accelerated growth to meet the predicted higher demand for wood-based products and to increase forest health. Traditional forest tree breeding has been used to obtain better growing and more resistant trees, but it is inherently limited by the pace of natural selection and existing genetic variation. New genomic techniques, particularly with the advent of CRISPR technologies, provide a powerful toolkit for introducing targeted genetic modifications, circumventing these limitations [70]. Moreover, CRISPR-based genome editing can significantly reduce the time required to develop trees with desired traits, a crucial advantage given their several decades of growth to full maturity [71]. Crucially, for long-lived species like forest trees, engineered or bred resistance must be durable, persisting even under conditions favorable to pathogens or pests [72,73]. This section will delve into the potential applications of CRIPSR technologies for addressing key challenges and enhancing desirable traits in forest tree improvement (Figure 2), focusing on four key areas: (1) enhanced resistance to biotic stressors; (2) abiotic stress tolerance; (3) improved wood properties; and (4) accelerated growth and biomass production.

### 3.1. Enhanced Resistance to Biotic Stressors

A central challenge in forest biotechnology is engineering durable resistance to pathogens, ensuring the long-term health and productivity of these vital ecosystems. CRISPR technologies have been developed to explore effective ways for engineering pathogen resistance, either by (1) precisely improving host factors that are involved in the defense mechanisms, or (2) directly targeting and destroying invading pathogens using pathogen-inducible CRISPR-mediated genome-editing systems [74]. The latter approach is restricted to confer resistance against RNA and DNA viruses by transforming the plant genome to express a CRISPR/Cas system together with a gRNA targeting a locus or multiple loci in the genome of the virus [74,75,76]. For targeting of DNA viruses, the Cas9 effector is used [75], whereas for targeting RNA viruses, the Cas13 system is used [76,77,78,79]. Due to the transgenic nature of the method, it is not yet feasible for commercial applications in most parts of the world, and to our knowledge, the only application in a tree was shown by Spencer and colleagues to induce resistance to Grapevine Virus A [79]. The former approach, on the other hand, targets specific loci in the plant genome. Developing resistance in plants can often be achieved either through integration of resistance (R) genes or through the disruption of susceptibility (S) genes [56,80]. For this, a detailed understanding of the host–pathogen interaction and response pathways is necessary [72]. In annual model plants, research of such interactions and pathways has already been established and many important genes have been identified and their function has been described. However, this knowledge can only be cautiously used in forest trees since the perennial and long living forest trees have a higher number of biotic and abiotic stress resistance genes [70,81] and also the pathways that are involved can differ.

#### 3.1.1. Targeting Susceptibility Genes: Disrupting Pathways That Facilitate Infection

Susceptibility (S) genes, which are responsible for increased susceptibility of the plant to certain pathogens, are a good target for genome editing and are advantageous compared to the introduction of R genes because S gene targeting often provides more durable resistance and can be non-race-specific [56,82]. S genes can be grouped into two categories: (i) genes acting as a negative regulator to plant immunity and (ii) genes that are targeted by pathogen effector molecules [74,83]. CRISPR/Cas systems can be utilized for indel creation and thus for disruption of the S gene, which renders the plant more resistant to the pathogen [83]. To overcome this type of resistance, the pathogen must acquire new functions, a process that is generally thought to be more difficult. This, in turn, facilitates a durable resistance [82]. In addition, the use of multiplex CRISPR/Cas to target multiple S genes also has the potential to create an even more durable resistance. However, since many S genes are involved in regulating important pathways or are linked to genes important for plant growth and development, editing of these can lead to a decrease in fitness and undesirable phenotypes [80]. To overcome these problems, a solution could be to preserve the physiological function of the S gene product but disrupt its recognition by the pathogen by altering the amino acid sequence through genetic engineering, such as base or prime editing [80,84]. Alternatively, the editing of the *cis* regulatory elements might be a possible solution [85]. For example, in various fruit trees, S genes have already been edited using CRISPR/Cas9 for InDel creation; edited plants often showed higher resistance (Apple [86,87,88]; Cacao [89]; Citrus [90]). In poplar, Yao and colleagues used CBE to create an immature stop codon in a transcription factor involved in the response of the plant to fungi [61]. These studies are a proof of concept and show the first results that targeting susceptibility factors using CRISPR/Cas is a useful method for inducing plant resistance; however, longer field tests need to be carried out to determine other phenotypic effects and how durable the resistance is in natural (non-lab) conditions.

#### 3.1.2. Utilizing Resistance Genes: Enhancing Innate Defense Mechanisms

Resistance (R) genes mostly provide full or partial resistance of a host to a pathogen by recognition of pathogen effector molecules, either on the cell surface or in the cell [91]. After recognition of a pathogen effector molecule, they usually start an effector-triggered immunity, which often leads to a hypersensitive response, i.e., often cell death [91]. Over the past three decades, more than 450 R genes have been identified, highlighting their importance in plant immunity systems [92]. However, our understanding of natural variation in R genes within diverse tree species, particularly at the protein structure and functional level, remains limited. Exploring this natural diversity represents a considerable untapped resource for engineering disease resistance.

Traditional breeding, transgenic approaches, and now genome editing can be used to introduce, modify, or stack R genes to confer resistance to a particular pathogen [93,94]. However, relying on a single R gene often leads to resistance that is not durable, as pathogens can rapidly evolve to overcome recognition [95]. Pyramiding multiple R genes, an approach that combines multiple resistance genes within a single plant, offers a promising strategy for achieving more durable resistance [96,97].

Beyond simply introducing or pyramiding R genes, fine-tuning their regulation and expression patterns is crucial for optimizing the plant defense response. Novel molecular switches, such as those controlling pathogen-induced translation [98,99,100], can be harnessed with CRISPR technology to precisely regulate the expression of R genes and master immune regulators like Nonexpressor of Pathogenesis-Related genes (*NPR* genes). For example, while *NPR1* is an essential component for SA-mediated systemic acquired resistance [101,102], its homologues *NPR3/4* are SA receptors [103] that negatively regulate *PR* gene expression and SA defenses [104,105]. Recently, downregulating or knocking out *NPR3* in *Arabidopsis* [106], potato [107], and cacao (*Theobroma cacao*, [89]) enhanced resistance to bacterial and fungal pathogens by activating SA-mediated defenses and JA catabolism.

### 3.2. Climate-Proofing Forests: CRISPR Equips Trees with Enhanced Abiotic Stress Tolerance

While every tree species possesses an inherent capacity to adapt to environmental change, the rapid pace of current climate change poses a significant threat to forest health and biodiversity in many parts of the world [108]. Natural adaptation, driven by selection processes, unfolds over millennia—a timeframe far exceeding the urgency of the climate crisis.

For instance, the natural range shifts of tree species following past climatic events, such as the northward migration of the pedunculate oak (*Quercus robur*) after the last ice age (10,000 years ago), provide valuable clues about adaptive genetic variation [109,110,111,112]. Deciphering the genetic basis of these local adaptations can reveal potential targets for CRISPR-mediated enhancement of stress tolerance in closely related tree species.

Furthermore, our understanding of plant stress responses has been dramatically accelerated by studies in model plants and CRISPR-edited crops [113,114]. These studies have unveiled intricate regulatory networks governing drought and salinity tolerance, two critical traits for climate resilience.

CRISPR is now being harnessed to translate this knowledge into actionable solutions for forest trees. By introducing targeted modifications in key genes and pathways, researchers can enhance drought and salinity tolerance, bolstering the resilience of these vital ecosystems. For example, CRISPR-mediated knockout of the transcription factor gene *WRKY77*, a negative regulator of salt tolerance, significantly improved the salt tolerance in *Populus alba* var. *pyramidalis* under in vitro conditions [115].

Further CRISPR-based studies in poplar have provided a detailed understanding of drought resistance mechanisms, paving the way for refined breeding approaches. These studies have implicated various transcription factors in regulating key stress responses, including root growth (*NF-YB21* [115]), stomatal control (*PdGNC* [116], *OSIC1* [117]), water potential maintenance (*PtoERF15* [118]), and gibberellin modulation (*PtoMYB142* [119]).

CRISPR-based approaches for enhancing abiotic stress tolerance hold immense potential for climate-proofing forests, ensuring their continued provision of essential ecological, economic, and social services in a rapidly changing world. However, it is crucial to consider potential trade-offs between enhanced stress tolerance and other important traits [120], such as growth and wood quality, during the development of climate-resilient trees.

### 3.3. Engineering the “Perfect” Timber: CRISPR-Enabled Tailoring of Wood Properties

Wood, a remarkable renewable biomass resource, plays a vital role in countless industries, from construction and paper production to biofuel generation and chemical synthesis. Its versatility stems from its unique chemical composition and intricate cell wall structure, both of which can be precisely edited using CRISPR technology to meet specific application demands [33,35]. One of the most promising avenues for wood improvement lies in manipulating lignin biosynthesis. Lignin, a complex polymer that provides structural rigidity to wood, also poses challenges for efficient processing in pulp and paper production. CRISPR-mediated genome editing has emerged as a powerful tool for fine-tuning lignin content and composition, enhancing wood processability and unlocking its full potential [31,121].

Early CRISPR studies in poplar, targeting specific genes involved in lignin biosynthesis, such as lignin-associated *4-coumarate:CoA ligases 4CL1* [122], *caffeoyl shikimate esterase CSE1/2* [123,124] and *cinnamoyl CoA reductase CCR2* [125,126], have demonstrated the feasibility of this approach, leading to improved wood processing efficiency. In addition, CRISPR-based editing of the laccase gene *LAC14* [127], which oxidizes monolignols to initiate their coupling to lignin or of the acyltransferase *PHBMT1* [128], which mediates p-hydroxbenzoylation of monolignols, also led to a reduction in lignin or, respectively, tailored lignin physiochemical properties for the improved delignification of woody biomass in the pulping process. However, these cell wall lignin modifications, typically focusing on single or double gene knockouts, often neglected the complex, multigenic nature of monolignol biosynthesis, which involves at least 21 genes in poplar [129,130]. To optimize multiplex CRISPR strategies for lignin modification, Sulis and colleagues leveraged a computational predictive model [131] integrating transcriptomic, proteomic, fluxomic, and phenomic data from thousands of transgenic poplar lines [130]. By systematically exploring potential gene combinations, they identified the most promising targets for enhancing fiber traits [132]. Rationally designed multiplex CRISPR editing, targeting three to six lignin biosynthesis genes, resulted in trees with reduced lignin content, increased lignin monomer syringyl-to-guaiacyl (S/G) ratio, and enhanced carbohydrate-to-lignin (C/L) ratio, leading to higher pulp yields in micropulping experiments. However, many 6-month-old low-lignin lines also exhibited reduced tree growth and stem volume, demanding additional studies to establish the impact of such a change in long-term field environments.

### 3.4. Accelerated Growth and Biomass Production: Meeting Demands for Renewable Resources

As the demand of renewable resources continues to surge, enhancing the growth and biomass production of forest trees becomes increasingly critical. Tree growth, a complex symphony orchestrated by a myriad of genetic and epigenetic factors, is intricately linked to both forest ecosystems and planted forest productivity [24,112,133]. The symphony of growth unfolds through a carefully choreographed sequence of events, starting with cell division and expansion in the apical and cambial meristems. Developmental and seasonal transitions, photosynthetic efficiency, nutrient and water uptake, and the ability to respond to biotic and abiotic stresses all play vital roles in determining overall tree growth [134,135]. This intricate interplay of processes is reflected in the genetic architecture of growth traits, often characterized by a large number of QTLs with small individual effects [112,136,137,138]. This complexity has made it challenging to pinpoint key genetic levers for manipulating growth.

High-throughput, multiplex targeting of CRISPR technology offers a powerful new tool for dissecting this complexity and accelerating the development of fast-growing, high-yielding trees in short rotation plantations. Recently, CRISPR-based studies in woody plants have provided valuable insights into the genetic control of tree growth and tree architecture, laying the groundwork for future breakthroughs [139]. For example, CRISPR-mediated knockout of the *Arabidopsis thaliana* BRANCHED1 orthologs (*PcBRC1-1* and *PcBRC2-1*) in *Populus* × *canescens* led to a dramatic increase in bud outgrowth, highlighting the potential for manipulating branching patterns to enhance biomass production [140,141]. In another study, targeting the rice ortholog TILLER ANGLE CONTROL 1 (*TAC1*) in *P.* × *canescens* resulted in a more upright growth habit, though without a significant increase in biomass [142].

In the last few decades, by using functional genomic approaches, including CRISPR-based tools, we began to understand the genetic mechanisms that control annual growth and dormancy cycles in perennial plants, including the perception, signaling and response to seasonal changes. For instance, in a recent study in poplars, Ding et al. found that the phytochrome B (phyB)-PHYTOCHROME INTERACTING FACTOR (*PIF*) regulon plays a crucial role in two major phenological transitions: bud flush and bud set [143]. These transitions are primarily cued by accumulated temperature units and photoperiod, respectively. CRISPR-ed knockout mutants of a Populus PHYB paralog (*PHYB2*) displayed strikingly increased height growth, despite a shorter growing season. The study also showed that the PHYB1 paralog can partially compensate for the loss of PHYB2 activity, as a double-knockout mutant (PHYB1B2KO) exhibited a strong growth cessation phenotype [143]. Furthering our understanding of seasonal growth regulation, Gao et al. demonstrated that an ortholog of the photomorphogenesis regulatory factor ELONGATED HYPOCOTYL 5 (HY5a) in *Populus tomentosa* modulates seasonal growth. HY5a acts by regulating the PHYB2-HY5a-FT2 (FLOWERING LOCUS T2) module to control the onset of winter dormancy, and by fine-tuning gibberellin (GA) levels to influence bud-break [144]. Intriguingly, *HY5a* knockout lines exhibited increased plant height, larger leaf lamina size, and greater biomass compared to the wide type. These findings suggest that manipulating *HY5a* could be a promising avenue for optimizing tree adaptation to new environments, enhancing tree survival and productivity.

Looking ahead, CRISPR-based genome editing holds immense promise for enhancing forest productivity. Future studies should focus on validating promising candidate genes identified through GWAS, QTL, and Genomic Selection analyses [112,145,146,147] with the assistance of huge computational power and new artificial intelligence and machine learning tools [148,149] of growth-related traits in tree species. By fine-tuning the expression of key genes involved in growth regulation, photosynthesis, and resource allocation, CRISPR could promote a new era of high-yielding, sustainable plantation forestry intended for biomass production.

## 4. Challenges and Opportunities in Forest Tree Genome Editing

### 4.1. Reagent Delivery: Overcoming Barriers in Forest Tree Cells

Efficient and rapid integration of the required gRNAs and Cas proteins into the plant cell is one of the major bottlenecks for CRISPR/Cas-mediated gene editing in forest trees. Due to the rigid cell wall and often high genome complexity of plant cells, delivery of gene editing reagents is challenging [150]. For delivery of the editing proteins and gRNAs, different methods are available depending on the type (DNA, RNA or protein) and size of the cargo, targeted species, targeted tissue and whether integration of transgenes into the genome is intended [151]. Utilization of the plant-associated bacterium *Agrobacterium tumefaciens* to integrate an engineered DNA-sequence present on the Ti-plasmid into the plant genome is one of the most used methods for CRISPR/Cas reagent delivery [151]. Many trees, however, are recalcitrant to *A. tumefaciens*-mediated delivery, or regeneration of the transformed plants takes a lot of time [151]. Also often used is particle bombardment, which overcomes bottlenecks of *A. tumefaciens,* such as a limited host range, but it can cause unwanted chromosomal rearrangement [150,151].

Avoidance of transgene integration can be important, as CRISPR/Cas-edited plants that lack integrated transgenes do not fall under the genetically modified organism regulations in many countries [152]. Recently, DNA-coated gold microparticles have been used to deliver DNA encoding CRISPR/Cas9 and sgRNA into hybrid poplar calli. Through transient expression of the CRISPR/Cas9 system and an antibiotic resistance gene for selection, biallelic edits were produced without integration of transgenes, albeit only at a low efficiency [153]. Another approach for avoiding the integration of transgenes is the delivery of ribonucleoproteins (RNPs), consisting of preassembled Cas nuclease proteins and gRNA (Cas-gRNA complex), into protoplasts [150]. In the rubber tree, RNPs were delivered into protoplasts using polyethylene glycol-mediated transfection and achieved targeted mutation frequencies of 3.74% to 20.11% across five targeted loci [154]. In apple and grapevine, this approach was also used and resulted in varying mutation frequencies dependent on the ratio of sgRNA and CRISPR/Cas proteins [86]. In forest trees, RNPs have also been successfully delivered into cells, for example, into somatic embryogenesis tissue of *Pinus radiata*, which produced only monoallelic edits [155]. In addition, RNP delivery into *Castanea sativa* protoplasts led to editing efficiencies of up to 21.4% [156]. Unfortunately, regeneration of the edited protoplast is difficult and for forest trees, regeneration protocols are available only for a few species [31]. For those species, such as conifers (including Taxaceae), poplars, cork oak (*Quercus suber* L.), *Jatropha curcas*, *Betula pendula*, etc., which already employ anther culture and microspore embryogenesis, pollen-based delivery methods such as HI-EDIT [157] might offer an efficient route to transgene-free homologous gene-edited plants. Other transgene-free delivery methods currently tested in model and crop species that could also be of benefit include the use of viral vectors, which facilitate delivery of gene editing reagents without the use of tissue culture for plant regeneration, but are currently restricted by limited cargo size [151]. Recently, the delivery of RNPs using nanoparticles has been demonstrated in citrus protoplast cells [158,159], offering an additional non-transgenic platform for genome editing in tree species [151,160,161]. Remarkably, by fusing tRNA-like structures to mRNA to enhance transcript mobility, Yang et al. have demonstrated the movement of recombinant Cas mRNA and gRNAs containing tRNA-like structures from transgenic rootstocks to non-transgenic scions through graft junctions [162]. This movement enables heritable gene editing in the scion, ultimately resulting in transgene-free, gene-edited progeny. This innovative approach highlights the dynamic progress being made in CRISPR delivery methods for plants. While a universally applicable, tissue-culture-free protocol for delivering CRISPR reagents remains elusive, the diverse array of approaches currently being explored and tailored holds promise for overcoming delivery challenges in a wide range of tree species.

### 4.2. Regeneration of Edited Plants: Efficient and Genotype-Independent Regeneration Protocols

As of right now, many methods still rely on tissue culture to regenerate plants from the edited cells, which, for some plant species, have not yet been established and often take a long time [163]. Higher efficiency and less species and genotype restrictions for regeneration of edited cells is therefore another major bottleneck. Recent advances in our understanding of plant developmental regulators offer promising solutions. For example, *BABY BOOM* (*BBM*, [164]), *WUSCHEL* (*WUS*, [165]), *WUSCHEL-related HOMEOBOX 5* (*WOX5*, [166]), the chimeric growth factor of *GROWTH-regulating factor* (*GRF*) and *GRF-interacting factor* (*GIF*) [167], several *DNA binding with one finger* (*DOF*) transcription factors [168], and, very recently, a plant elicitor peptide *REGENERATION FACTOR1 (REF1)* [169] have been utilized to significantly boost plant regeneration efficiency and also promote the regeneration of otherwise recalcitrant genotypes. Transferring these advancements to forest biotechnology could enable editing in previously challenging species, accelerating the development of improved trees.

Furthermore, CRISPR-Combo, a system developed by Pan and colleagues, allows for simultaneous gene editing and transcriptional regulation, which can boost the regeneration ability of edited plants [170]. This system leverages the length of the protospacer to control the activity of the CRISPR/Cas system: protospacers between 17 and 20 nucleotides induced mutagenesis, while those with 14–16 nucleotides mediated gene activation [170]. By using either a Cas9-activation system fusion or a BE-activation system fusion, Pan et al. can target the locus for editing using a longer protospacer and the locus for activation with a shorter protospacer [170]. This design ensures that both processes can occur without interference. The versatility of CRISPR-Combo was demonstrated by simultaneously base-editing a poplar gene and activating endogenous morphogenesis genes (i.e., *WUS* and *WOX11*), leading to a faster and more efficient regeneration of poplar plants [170]. Thus, CRISPR-Combo provides a powerful approach to overcome the regeneration barrier of recalcitrant tree species by, for example, simultaneous activation of multiple master developmental regulators and essential morphogenic genes.

### 4.3. Muliplex Editing: Targeting Multiple Genes for Complex Trait Engineering

The ability to edit multiple loci can be of great benefit for controlling important traits of forest trees, since many traits (especially yield- or stress resistance-related) are highly polygenic [25]. Sulis and colleagues used a multiplex CRISPR/Cas9 approach to simultaneously edit up to six genes involved in lignin biosynthesis, which led to a higher decrease in lignin content compared with single gene editing approaches, thereby enhancing poplar wood for better fiber pulping and reduced carbon emissions [132]. CRISPR/Cas systems are well suited for multiplex editing, as just different gRNAs targeting different loci need to be expressed in the cell, which then bind to the Cas-effector [171,172]. However, to unleash the full potential of multiplex editing, challenges still need to be tackled. When expressing a higher number of gRNAs, more gRNAs are competing to bind with the Cas effector, leading to lower editing efficiencies for the gRNAs, a phenomenon called retroactivity [173,174]. Furthermore, the creation of multiple DSBs in the genome can lead to unintended chromosomal rearrangements [174,175,176]. By targeting multiple loci, the chance for off-target effects also increases [174]. The location of the gRNA in the RNA cassette also affects efficiency of the specific gRNA [174].

## 5. Expanding the CRISPR Toolbox Beyond Genome Editing of Forest Trees

Beyond its transformative potential for genome editing, CRISPR technology is expanding its reach within forestry, offering a diverse toolkit for diagnostics, deciphering pathogen virulence, functional genomics, direct evolution, and population-level genetic manipulation. This section explores these emerging applications of CRISPR, highlighting their potential contributions to forest health, biodiversity conservation, and sustainable biotechnology.

### 5.1. CRISPR-Based Diagnostics: Revolutionizing Pathogen and Pest Detection in Forestry

CRISPR/Cas systems are rapidly transforming nucleic acid detection, offering a powerful alternative to traditional methods for identifying pathogens and pests in forestry. Leveraging the targeted nuclease activity of Cas enzymes, CRISPR-based diagnostics provide exceptional sensitivity, specificity, portability, and speed, enabling rapid and accurate detection of diverse DNA and RNA targets [177,178,179].

Pioneering platforms like DETECTR [180] and SHERLOCK [181,182] harness the collateral cleavage activity of Cas12 and Cas13 orthologs, respectively, achieving attomole sensitivity (10^−18^ mol/L) in detecting multiple DNA and RNA targets. While preamplification using qPCR or NGS remains the gold standard for sensitive detection, these methods are often constrained by the need for specialized equipment and complex workflows. However, recent advancements have integrated CRISPR with isothermal amplification technologies (IATs), such as recombinase polymerase amplification (RPA), recombinase-aided amplification (RAA), loop-mediated isothermal amplification (LAMP), and rolling circle amplification (RCA), enabling ultrasensitive detection of nucleic acids without thermal cyclers.

This has paved the way for the development of rapid, field-deployable CRISPR-based diagnostics for plant pathogens, exemplified by successful detection of multiple RNA apple viruses/viroids [183], Citrus Tristeza Virus [184], *Phytophthora nicotianae* oomycete causing crown rots and wilt disease in a wide host range, including tree species [185], peach Brown rot fungus *Monilinia fructicola* [186], and phloem-limited, fastidious, gram-negative bacteria ‘*Candidatus* Liberibacter asiaticus’, causing citrus greening disease [187]). Portable CRISPR-based “field kits” hold immense promise for immediate and efficient disease and pest management in forestry.

Beyond plant pathogens, CRISPR-based diagnostics have demonstrated potential in detecting invasive insect pests [188]. A recent study successfully combined RPA with CRISPR to identify three globally invasive moth species (*Keiferia lycopersicella*, *Phthorimaea absoluta* and *Scrobipalpa atriplicella*) [188]. Furthermore, rapid visual detection assays have been developed for quarantine pests such as guava fruit fly (*Bactrocera correcta*, [189]), Khapra beetle (*Trogoderma granarium* Everts, [190]), highlighting the potential of CRISPR in managing forest insect invasions.

### 5.2. Deciphering Pathogen Virulence: CRISPR as a Tool for Understanding and Disarming Forest Foes

By enabling precise genome editing in a wide range of fungal and fungal-like oomycete species, CRISPR technology has become a powerful tool for dissecting the intricate interplay between fungal(-like) pathogens and their hosts, providing unprecedented insights into virulence mechanisms and paving the way for novel and targeted disease control strategies (Figure 2) [191]. This is particularly valuable for studying oomycetes, a group of notorious pathogens that cause devastating damage to plants, including those in forest ecosystems [192,193]. Oomycetes have shown to be exceptionally difficult to manipulate using traditional genetic approaches because of their complex life cycles, low rates of homologous recombination and highly heterozygous genomes [193].

One key application of CRISPR in plant pathogen research is the identification and characterization of virulence genes. For example, CRISPR/Cas9 knockout of an avirulence gene (*AvrLM7*) in *Leptosphaeria maculans* (the causal fungal agent of blackleg disease on *Brassica* spp.) revealed its role in evading host recognition and enabling infection of previously resistant *Brassica napus* cultivars (e.g., canola plants with the *Rlm7* resistance gene) [194]. Further comparative pathogenicity tests of the mutated *AvrLM7* gene on a collection of canola genotypes allows identification of potential targets for developing durable resistance in canola [194].

Furthermore, CRISPR-mediated genome editing has enabled the targeted disruption of essential pathogenicity factors, effectively disarming these foes. Studies in *Phytophtora* oomycete species, notorious for causing devastating diseases in various tree species, have demonstrated the potential of this approach [195]. Disrupting an extracellular cysteine protease inhibitor (*PpalEPIC8*) in *Phytophtora palmivora* significantly reduced its virulence on papaya trees [196]. Similarly, knocking out a gene (*Ppal15kDa*) encoding a secreted glycoprotein crucial for appressorium formation in *P. palmivora* led to the loss of nearly all pathogenicity to papaya species [197].

The complex web of fungal interactions in forest ecosystems, encompassing saprotrophs, symbionts, pathogens, and endophytes [198], provides a unique opportunity to harness CRISPR beyond simply targeting pathogens. By manipulating the genomes of beneficial fungal species, one could enhance their natural antagonistic properties, boosting their ability to suppress pathogenic fungi and therefore bolster tree resistance [192].

### 5.3. Engineering the Microbiome: Promoting Beneficial Microbial Interactions

Forests trees are hosts for a wide range of microbial organisms that live either on the surface or inside of the tree tissues. The nature of these plant–microbe relationships can be either mutualistic, commensalistic, parasitic or pathogenic [199,200,201]. Thus, the microbiome can impact forest health and resistance of the trees to diseases and abiotic stresses [201] and research has shown a linkage between the plants’ microbiome and resistance to diseases [70,202]. Multiple interactions have been identified through which the microbiome can confer resistance to a disease. The microbiome influences the physiology of the plant, which impacts pathogens and is responsible for induced systemic resistance [70,203,204]. Also, the microbiome can influence invading pathogens and pests through competition, exclusion and antibiosis and change the nutritional status of the plant [70]. Especially for endophyte composition, the host genotype is one of the main factors responsible [201]. Changes in the plant genome can therefore change the composition of the microbiome and thus also its interaction with pathogens and pests (Figure 2). In poplar, the silencing of an important lignin biosynthesis gene led to changes in the endophytic microbiome composition [205]. In general, induced mutations do lead to changes in the endophytic microbiome composition, but not to changes in soil microbiome [206,207]. However, before such precise changes can be made, a detailed understanding of the plant–microbe interaction at the genetic level is necessary [206]. In addition to understanding the plant–microbe interaction, it is also important to know the full function of a gene to prevent other undesirable phenotypic events.

### 5.4. Functional Genomics and Fine-Tuning Gene Regulation

CRISPR/Cas systems are well suited for functional genomics studies due to their site specificity and ability to target multiple genomic loci by introducing multiple sgRNAs. In recent years, many forest tree genomes have been sequenced; however, except for the model tree poplar, only a few studies dissecting the function of the sequenced genes have been performed [29]. Unravelling the function of genes is a big step towards more efficient tree breeding and genetic engineering. For functional genomics studies, two approaches are often used, loss-of-function and gain-of-function approaches (Figure 2) [208]. For loss-of-function mutations, CRISPR/Cas9 can be used to induce InDel creation through NHEJ of the DSB, which often leads to gene knock-out [208]. Therefore, mostly constitutively spliced exons are targeted, which leads to frameshift mutations [208]. Alternatively, CRISPR-mediated inhibition (CRISPRi) can be used to downregulate expression of target genes. In most cases, CRISPRi consists of a dCas9 fused to a transcriptional repressor domain and works by interfering with the binding of transcriptional regulators, the binding of RNA polymerases, or by interfering with transcriptional elongation [174,209,210]. Gain-of-function can be achieved by using CRISPR-mediated activation (CRISPRa), which involves fusing a dCas9 to a transcriptional activator domain, to increase expression of the target gene [174]. Such approaches work especially well when multiplexing multiple sgRNAs that either target a single locus to increase effectiveness of the inhibition/activation or when multiplexing sgRNAs to target multiple genes, allowing for modulation of metabolic pathways [174]. In trees, many functional genomics studies using CRISPR/Cas systems have already been conducted and have led to the dissection of the genetic basis for various traits [30,211]. However, most of them have been carried out either in the model tree poplar or fruit trees, leaving especially gymnosperms, the main tree group in many forests of the world, still understudied.

### 5.5. Directed Evolution: Accelerate the Evolution of Desirable Traits

To enhance or modify the function of genes of interest, directed evolution (DE) can be used [56]. DE works by generating genetic variation by inducing mutations and then selecting the desired genotypes under selective pressure to obtain individuals with the desired traits (Figure 2) [56]. CRISPR/Cas made this approach more efficient than earlier methods using PCR or DNA shuffling [55]. Most DE systems work by using base editors in combination with a sgRNA library that contains sgRNAs spanning the sequence of interest both on the forward and reverse DNA strand [56,212]. Another way is to use CRISPR/Cas9 with a sgRNA library to create mutations through NHEJ, which has been deployed to confer resistance to splicing inhibitors [213]. Kuang and colleagues developed the base-editing mediated gene evolution (BEMGE) method that employs CBE and ABE effectors, each with a sgRNA library spanning the length of the gene coding sequence [212]. This method has been used to evolve the rice gene *OsALS1* to induce resistance to the herbicide bispyribac-sodium [212]. A system termed saturated targeted endogenous mutagenesis editors (STEME) that consists of a fusion of cytidine deaminases and adenosine deaminases bound to an nCas9 with an uracil DNA glycosylase inhibitor was also developed [55]. Li et al. also tested another variant with a nCas9 recognizing NG PAM sequences (STEME-NG). Both variants worked for inducing mutations and were more efficient than one CBE and one ABE expressed simultaneously [55]. They tested the editors on the *Oryza sativa OsACC* gene and showed that both induced herbicide resistance through edits in the coding sequence of the gene [55]. These studies show that using CRISPR/Cas-mediated directed evolution in plants is already feasible and developed systems that can now also be tested and optimized in other plant species. However, until now, the approach has only been used for herbicide resistance; to use it for the enhancement of other properties, new selection methods need to be developed [56]. Since, in trees, it can take a long time until phenotypic properties can certainly be observed, such directed evolution approaches can be time-consuming in tree breeding applications.

### 5.6. CRISPR Gene Drive and Genetic Containment: Population-Scale Genomic Alterations

CRISPR gene drive technology offers a revolutionary approach to manipulating entire populations by rapidly spreading CRISPRed genes. This technology exploits biased inheritance mechanisms, ensuring that the CRISPRed gene is inherited by almost all offspring, leading to its dissemination throughout the population. Imagine promoting the spread of climate resilience traits in endangered tree species, or even preemptively introducing “anticipatory” genetic defenses against likely future invasive pathogens; the possibilities seem limitless. The spread of favorable alleles still relies on gene flow or traditional breeding methods, which are inherently slow processes that often require many generations to reach high frequency in wild populations, especially for traits with modest fitness advantages. Furthermore, mass release of CRISPRed individuals can be impractical and may inadvertently increase the frequency of unintended, potentially harmful alleles.

Natural selfish genetic elements provide inspiration for gene drive systems. Transposable elements, notorious for their “jumping gene” behavior, and homing endonuclease genes (HEGs), which drive site-specific gene conversion events, are examples of naturally occurring gene drives [214]. Modern CRISPR-based homing gene drives typically consist of three components: (i) the Cas9 nuclease, (ii) an sgRNA targeting the desired genomic location, and (iii) homology arms flanking the target site to facilitate HDR [215]. The drive mechanism works by first inducing a DSB in the wild-type allele, which is then repaired using the gene drive-containing chromosome as a template, effectively converting the wild-type allele to a gene drive allele. This biased inheritance, shifting from Mendelian (50%) to super-Mendelian (>50%) transmission, allows the gene drive to rapidly spread through the population.

While this technology holds promise for applications like preventing disease transmission by insect vectors [216,217] or enhancing forest resilience against invasive pathogens, significant hurdles remain. The low efficiency of HDR, coupled with the need for effective germline-specific promoters to drive Cas9 expression, poses substantial challenges [218]. Compared with constitutive promoters, germline-specific promoters such as Arabidopsis *DD45* (egg cell and early embryo [219,220]), Yao (shoot apical and root meristem-active [221]), tomato *Lat52* (pollen [219]) and EC (egg cells, embryo [222]) can increase the frequency of heritable edits in progeny.

Recently, a novel cytotoxicity detoxification gene drive system inspired by a natural selfish genetic element in rice has demonstrated remarkable success in subverting Mendelian inheritance [223,224]. This system works by selectively killing non-drive-carrying offspring, ensuring the rapid spread of the drive allele. Inspired by this approach, synthetic CRISPR gene drives are being developed to manipulate plant populations by impairing pollen germination or eliminating gametes lacking the drive element [225,226].

However, the ecological and ethical implications of releasing self-propagating gene drives into the environment remain a topic of intense debate. Thorough risk assessment and robust regulatory frameworks are essential before deploying such powerful technology in natural ecosystems.

In contrast to gene drives, which aim for widespread genetic alterations, genetic containment strategies focus on preventing the spread of engineered genes to wild populations. CRISPR-based gene editing can be used to disrupt genes involved in pollen dispersal or create reproductive barriers, effectively limiting gene flow between modified trees and their wild relatives. For example, CRISPR-mediated disruption of the LEAFY ortholog in *Eucalyptus* demonstrated effective sexual containment without significant negative impacts on vegetative growth [227,228].

## 6. Conclusions and Future Perspectives

CRISPR technologies have emerged as a transformative force in biotechnology, offering an unprecedented toolkit for enhancing tree resilience, productivity, and adaptability in a rapidly changing world. The CRISPR systems encompass a diverse array of tools, enabling precise base editing, sequence insertion and deletion, and modulation of gene expression, with new advancements emerging at a rapid pace [51,229,230]. However, translating this technology into tangible benefits for forestry requires overcoming several key challenges. Efficient delivery of CRISPR reagents into tree cells, coupled with robust and reliable regeneration protocols for a wide range of forest tree species, remains a critical bottleneck. Promising advancements, such as nanomaterial- and RNP-based delivery methods, and innovative regeneration strategies like CRISPR-Combo, elicitor peptide *REF1*, and *GRF*-*GIF* chimera, offer potential solutions. However, the majority of these and other new technologies are currently optimized for mammalian cells or model plants, necessitating further development and validation for application in forest trees.

CRISPR’s power lies in its ability to precisely manipulate the complex genetic networks underlying key tree traits, offering a path towards climate-resilient and productive trees. By unraveling the functions of individual genes and dissecting intricate pathways, CRISPR can inform both traditional breeding programs and genetic engineering strategies. For example, CRISPR-mediated editing of susceptibility (S) genes has the potential to confer a durable resistance to certain pathogens, while directed evolution can accelerate the development of novel, favorable genetic variations [231]. Still, it is crucial to recognize the intricate polygenic nature of many eco-physiologically and economically important traits, and to carefully consider potential unintended consequences of editing one trait on other, seemingly unrelated traits.

While CRISPR technology holds immense potential for future forestry, it is crucial to recognize that laboratory or glasshouse studies often fail to fully capture the complexity of real-world forest ecosystems [232,233]. Therefore, extensive long-term field trials, spanning decades, are essential to validate the efficacy and safety of CRISPR-edited trees under diverse environmental conditions. Furthermore, such research is important for fostering public dialogue and building trust in this transformative technology.

Ultimately, CRISPR should not be viewed as a replacement for conventional breeding but rather as a powerful complementary tool for generating targeted genetic diversity [234]. While the large majority of forest lands and species will likely remain untouched by CRISPR in the foreseeable future, its primary applications will likely focus on intensively managed, short-rotation clonal plantations, where specific wood properties are tailored for industrial use [33,235].

However, CRISPR also holds incremental potential for contributing to the protection and restoration of wild or feral forests, even in the face of mounting environmental challenges. By embracing a balanced and cautious approach, informed by rigorous scientific research and open public dialogue, we can harness the power of CRISPR to shape a more resilient and sustainable future for forests worldwide.

## Abbreviations

4CL1	lignin-associated 4-coumarate:CoA ligase 1
ABE	Adenine Base Editor
AvrLM7	avirulence gene 7 in *Leptosphaeria maculans*
BBM	BABY BOOM
BE	Base Editor
BEMGE	Base-Editing Mediated Gene Evolution
Cas	CRISPR-associated protein
CBE	Cytosine Base Editor
CCR2	cinnamoyl CoA reductase 2
CRISPR	Clustered Regularly Interspaced Short Palindromic Repeats
CSE1/2	caffeoyl shikimate esterase 1 or 2
DD45	gene 45 expressed in egg cells and early embryos
DOF	DNA binding with one finger transcription factor
DSB	Double-Strand Break
EC	egg cell-secreted protein
FT2	Flowering locus T2
GIF	GRF-interacting factor
GRF	growth-regulating factor
gRNA	guide RNA
HDR	Homology-Directed Repair
HEGs	homing endonuclease genes
HY5a	Elongated hypocotyl 5
IATs	isothermal amplification technologies
InDels	insertions or deletions
JA	jasmonic acid
LAC14	laccase gene 14
LAMP	loop-mediated isothermal amplification
Lat52	Pollen-specific gene 52
LEAFY	transcription factor promoting floral cell fate
NF-YB21	NF-Y gene B21
NGS	Next-Generation Sequencing
NHEJ	Non-homologous End Joining
NPR	Nonexpressor of Pathogenesis-Related gene
OSIC1	Osmotic stress induced C2H2 gene 1
PAM	Protospacer Adjacent Motif
PcBRC1/2	BRANCHED gene 1 or 2 from *Populus × canescens*
PdGNC	GATA, nitrate-inducible, carbon-metabolism involved protein from *Populus deltoides*
PE	Prime Editing
pegRNA	Prime Editing guide RNA
PHBMT1	*p*-hydroxybenzoyl-CoA monolignol transferase 1
PHYB	phytochrome B
PIF	phytochrome interacting factor
PtoERF15	Ethylene response factor 15 from *Populus tomentosa*
PtoMYB142	R2R3 MYB transcription factor 142 from *Populus tomentosa*
QTLs	quantitative trait loci
RAA	recombinase-aided amplification
RCA	rolling circle amplification
REF1	regeneration factor 1
RNPs	ribonucleoproteins
RPA	recombinase polymerase amplification
RPA	Replication Protein A
SA	salicylic acid
sgRNA	single guide RNA
STEME	Saturated Targeted Endogenous Mutagenesis Editor
TAC1	Tiller Angle Control 1
WOX5	WUSCHEL-related HOMEOBOX 5
WRKY77	transcription factor gene 77 containing WRKY domain
WUS	WUSCHEL
yao	a conserved WD-repeat containing nucleolar protein found in Arabidopsis *yaozhe* mutant
